# First report of *Anaplasma platys* infection in red foxes (*Vulpes vulpes*) and molecular detection of *Ehrlichia canis* and *Leishmania infantum* in foxes from Portugal

**DOI:** 10.1186/s13071-015-0756-y

**Published:** 2015-03-23

**Authors:** Luís Cardoso, Matan Gilad, Helder CE Cortes, Yaarit Nachum-Biala, Ana Patrícia Lopes, Maria João Vila-Viçosa, Margarida Simões, Paula A Rodrigues, Gad Baneth

**Affiliations:** Department of Veterinary Sciences, School of Agrarian and Veterinary Sciences, Universidade de Trás-os-Montes e Alto Douro (UTAD), Vila Real, Portugal; Koret School of Veterinary Medicine, The Hebrew University of Jerusalem, Rehovot, Israel; Victor Caeiro Laboratory of Parasitology, Instituto de Ciências Agrárias e Ambientais Mediterrânicas (ICAAM), University of Évora, Évora, Portugal; Animal and Veterinary Research Centre, UTAD, Vila Real, Portugal

**Keywords:** *Anaplasma platys*, *Ehrlichia canis*, *Leishmania infantum*, Polymerase chain reaction, Portugal, Red foxes, Canine vector-borne diseases, *Vulpes vulpes*

## Abstract

**Background:**

The bacteria *Anaplasma platys* and *Ehrlichia canis* and the protozoan *Leishmania infantum* are vector-borne agents that cause canine vector-borne diseases, some of which are zoonotic. The present survey investigated the prevalence of *Anaplasma*, *Ehrlichia* and *Leishmania* in red foxes (*Vulpes vulpes*) from Portugal by molecular analysis, in order to evaluate the epidemiological role of these canids as reservoirs of infection.

**Methods:**

Blood and/or bone marrow samples were collected from 78 red foxes obtained in eight districts of northern, central and southern Portugal. Real-time polymerase chain reactions (PCR) amplified a 123 bp fragment of the 16S rRNA gene of *Anaplasma* spp. and *Ehrlichia* spp. and a 265 bp fragment of the *L. infantum* internal transcribed spacer one (ITS1) region of the rRNA operon evaluated by PCR-high resolution melt analysis (PCR-HRM), with sequencing of the DNA products. A phylogenetic analysis was carried out to compare these to other sequences from *Anaplasma* spp. and *Ehrlichia* spp. deposited in GenBank®.

**Results:**

*A. platys* was detected in 10 (14.5%) and *E. canis* in two (2.9%) out of 69 foxes; and *L. infantum* was detected in one (1.3%) of the 78 foxes. The prevalence of *A. platys* was significantly different from the prevalence of *E. canis* (*p*=0.016) and from that of *L. infantum* (*p*=0.002). No co-infections were found in any one of the 78 foxes. No statistically significant differences were found between the type of sample (blood and bone marrow), geographic regions (north/centre and south), age (<2 years and ≥2 years) and gender for any one of the agents.

**Conclusions:**

This is the first known report of *A. platys* in red foxes worldwide, as well as the first molecular evidence of *E. canis* in foxes from Portugal. The moderate prevalence of *A. platys* suggests that red foxes may play a role in the epidemiology of infection with this bacterium and serve as a reservoir for domestic dogs.

## Background

The intracellular bacteria *Anaplasma platys* and *Ehrlichia canis*, and the protozoan *Leishmania infantum* are among the diverse range of vector-borne agents that cause canine vector-borne diseases (CVBD) [1]. *A. platys* is presumed to be transmitted by the *Rhipicephalus sanguineus* group ticks (*R. sanguineus* sensu lato [2]), infects platelets and causes canine infectious cyclic thrombocytopenia [3,4]. Canine infections with *A. platys* are mostly subclinical but clinical signs including lymphadenomegaly and pale mucous membranes have been reported in domestic dogs [5]. Although its virulence is generally low, *A. platys* might play a role in co-infections with other vector-borne agents [6]. Molecular evidence of *A. platys* was reported in a veterinarian co-infected with *Bartonella henselae* and *Candidatus* Mycoplasma haematoparvum [7], in two seronegative humans living in Midwestern USA and also in their dog [8], and in two women from Venezuela [9].

In Europe, *E. canis* is the etiological agent of canine monocytic ehrlichiosis and its confirmed vectors are *R. sanguineus* ticks [3]. Dogs can be subclinically infected with *E. canis* or present a spectrum of clinical manifestations that may reach fatal illness [10]. Clinical signs often include lethargy, anorexia, weight loss, fever, epistaxis and other haemorrhagic disorders, pale mucous membranes and lymphadenomegaly [11]. *E. canis* can also infect cats and wild canids [12,13], and human infections of a specific *E. canis* strain have been reported from Venezuela, revealing a zoonotic potential [14].

Leishmaniosis due to *L. infantum* is a major zoonosis potentially fatal to dogs and humans, representing an important veterinary medical and public health problem [15]. Phlebotomine sand flies (*Phlebotomus* spp.) are vectors and domestic dogs the main reservoir of the protozoan [16]. In humans, visceral leishmaniosis is the most severe clinical syndrome resulting from infections with *L. infantum*, and in southern Europe it is observed mainly in children and immunocompromised adults [17]. Canine leishmaniosis is a systemic chronic disease whose clinical manifestations usually include lymphadenomegally, dermatitis, alopecia, cutaneous ulceration, onychogryphosis, lameness, anorexia, weight loss, cachexia, ocular lesions, epistaxis, anaemia, diarrhoea and renal failure [18,19]. In addition to dogs, wild canids such as red foxes (*Vulpes vulpes*) might serve as hosts for *L. infantum* [20].

By harboring vector-transmitted pathogens, wild canids may constitute a potential reservoir for CVBD, some of which are zoonotic diseases. Red foxes are the most abundant wild carnivore in Europe and, due to a good adaptation to human environments, are invading many urban areas [21]. In Portugal, *A. platys*, *E. canis* and *L. infantum* have been reported as agents of CVBD in dogs [22-24], but no data are available regarding infection with the two bacterial pathogens in red fox populations. The present survey aimed at investigating the prevalence of *Anaplasma*, *Ehrlichia* and *Leishmania* spp. in red foxes from Portugal, by means of molecular analysis, in order to evaluate their role in the epidemiology of these infections.

## Methods

### Foxes and samples

Seventy-five carcasses of apparently healthy wild red foxes shot during the official hunting season or killed on the road due to traffic accidents were obtained between November 2008 and March 2010. The animals came from the districts of Viana do Castelo (n=9), Bragança (n=13), Vila Real (n=20), Braga (n=3) and Porto (n=2), in northern; Aveiro (n=2), in central; and Évora (n=26), in southern Portugal. Two additional red foxes from the southern district of Setúbal and one from Bragança were presented alive to the Center for Wildlife Rehabilitation, Veterinary Hospital of the University of Trás-os-Montes e Alto Douro, whose ethical committee approved the study as complying with the Portuguese legislation for the protection of animals (Law no. 92/1995).

The fox carcasses were refrigerated at 4°C, for no more than 72 h, or kept frozen at –20°C and thawed before sampling. During necropsy, clotted blood was collected from the right atrium or chest cavity and bone marrow from a femur, with sterile equipment, and stored at –20°C until further processing. Blood from the jugular or cephalic veins of the three living foxes was collected into EDTA tubes and also kept under the same frozen conditions as above. DNA was extracted from blood and bone marrow samples with commercial purification kits (QIAamp® DNA Blood Mini and QIAamp® DNA Mini; Qiagen, Valencia, CA, USA), as previously described [25].

Of 52 foxes whose gender was observed, there were 23 females and 29 males; gender was not recorded for 26 of the 78 foxes. Age was determined by morphologic characteristics and teeth eruption pattern and wear [26] in 48 foxes and ranged from 1.0 to 7.5 years, with a median value of 2.5 years (interquartile range: 1.5-3.5). Fourteen foxes were classified as juveniles (less than 2 years of age) and 34 as adults (2 years or more) [27].

### DNA amplification and sequencing

A 123 bp fragment within the 16S gene of both *Anaplasma* spp. and *Ehrlichia* spp. was amplified by real-time PCR using primers E.c-16S fwd and E.c-16S rev (Table 1) as previously described [28,29]. PCR was performed in a total volume of 20 μl containing 5 μl DNA, 400 nM of each primer, 10 μl Maxima Hot Start PCR Master Mix (2x) (Thermo Scientific, Epsom, Surrey, UK), 50 μM of SYTO9 solution (Invitrogen, Carlsbad, CA, USA) and sterile DNase/RNase-free water (Sigma, St. Louis, MO, USA), using the Corbett Research Rotor-Gene 6000 cycler (Corbett Research Pty Ltd, Sydney, Australia). Initial denaturation for 5 min at 95°C was followed by 45 cycles of denaturation at 95°C for 15 s, annealing and extension at 60°C for 30 s, and final extension at 72°C for 30 s. Amplicons were subsequently subjected to a melt step with the temperature raised to 95°C for 10 s and then lowered to 60°C for 1 min. The temperature was then raised to 95°C at a rate of 1°C per second. Amplification and melt profiles were analyzed using the Rotor-gene 6000 series software (Corbett Research Pty Ltd., Sydney, Australia). Positive samples from this reaction were further analyzed by conventional PCR using primers EHR16SD and EHR16SR (Table 1), which amplify a 345-bp fragment of the 16S rRNA gene found in the genera *Anaplasma* and *Ehrlichia* [30]. The PCR was performed in a total volume of 25 μl using PCR-ready High Specificity mix (Syntezza Bioscience, Jerusalem, Israel) with 500 nM of each primers and sterile DNase/RNase-free water (Sigma, St. Louis, MO, USA). Amplification was performed using a programmable conventional thermocycler (Biometra, Göttingen, Germany). Initial denaturation was at 95°C for 5 min, followed by 35 cycles of denaturation at 95°C for 30 s, annealing and extension at 55°C for 30 s, and final extension at 72°C for 30 s. After the last cycle, the extension step was continued for a further 5 min. PCR products were electrophoresed on 1.5% agarose gels stained with ethidium bromide and checked under UV light for the size of amplified fragments by comparison to a 100 bp DNA molecular weight marker.

**Table 1:** **Targeted genes and list of primers used in this study**

**Pathogen**	**Gene**	**Primer**	**Size (bp)**	**Location in locus/gene**	**Fragment length (bp)**	**References**
*Anaplasma* spp./*Ehrlichia* spp.	16S	E.c-16S fwd	TCGCTATTAGATGAGCCTACGT	285-408	123	[28,29]
		E.c-16S rev	GAGTCTGGACCGTATCTCAGT			
*Anaplasma* spp./*Ehrlichia* spp.	16S	EHR16SD	TAGCACTCATCGTTTACAGC	525-870	345	[30]
		EHR16SR	GGTACCYACAGAAGAAGTCC			
*Leishmania* spp.	ITS	ITS-219F	AGCTGGATCATTTTCCGATG	219-484	265	[31]
		ITS-219R	ATCGCGACACGTTATGTGAG			

A 265 bp fragment of the *Leishmania* internal transcribed spacer 1 (ITS1) region of the *L. infantum* rRNA operon was amplified by real-time polymerase chain reaction (PCR) using primers ITS-219 F and ITS-219R (Table 1) and then evaluated by high resolution melt (HRM) analysis [31]. The PCR was performed in a total volume of 20 μl containing 5 μl DNA, 200 nM of each primer, 10 μl Maxima Hot Start PCR Master Mix (2x) (Thermo Scientific, Epsom, Surrey, UK), 50 μM of SYTO9 solution (Invitrogen, Carlsbad, CA, USA) and sterile DNase/RNase-free water (Sigma, St. Louis, MO, USA), using the Corbett Research Rotor-Gene 6000 cycler (Corbett Research Pty Ltd, Sydney, Australia). Initial denaturation for 5 min at 95°C was followed by 50 cycles of denaturation at 95°C for 5 s, annealing and extension at 59°C for 30 s, and final extension at 76°C for 10 s. Amplicons were subsequently subjected to a HRM step with the temperature raised to 95°C for 10 s and then lowered to 60°C for 1 min. The temperature was then raised to 95°C at a rate of 0.3°C per second. Amplification and HRM profiles were analyzed using the Rotor-gene 6000 series software (Corbett Research Pty Ltd., Sydney, Australia).

DNA samples extracted from cell cultures of *L. infantum*, *Leishmania tropica*, *Leishmania major* and *E. canis* were used as positive controls for each corresponding PCR reaction and DNA from colony-bred dogs negative by PCR for vector-borne pathogens was used as negative control. A non-template control (NTC) with the same reagents described above but without DNA was added to each PCR to rule out contamination.

All positive PCR products were sequenced using the BigDye Terminator v3.1 Cycle Sequencing Kit and an ABI PRISM 3100 Genetic Analyzer (Applied Biosystems, Foster City, CA, USA), at the Center for Genomic Technologies, Hebrew University of Jerusalem, Israel. DNA sequences were evaluated with the ChromasPro software version 2. 1.1 (Technelysium Pty Ltd., Australia) and compared for similarity with sequences available in GenBank®, using BLAST program (http://www.ncbi.nlm.nih.gov/BLAST/).

### Phylogenetic analysis

A phylogenetic analysis, which included DNA sequences obtained from foxes from this study, was carried out to compare these sequences to other sequences from *Anaplasma* spp. and *Ehrlichia* spp. that had previously been deposited in GenBank®. Phylogenetic and molecular evolutionary analyses were conducted using MEGA version 6.06 (http://www.megasoftware.net) [32] and a phylogenetic tree was constructed by the Maximum-Likelihood algorithms using the Kimura-2-Parameter model. Bootstrap replicates were performed to estimate the node reliability, and values were obtained from 1000 randomly selected samples of the aligned sequence data.

### Statistical analysis

The Chi-squared or Fisher’s exact tests were used to compare proportions of positive results by gender and age group (juvenile vs. adults). McNemar’s test compared proportions in paired results, i.e. data obtained from blood and bone marrow of the same fox. A *p* value < 0.05 was considered as statistically significant [33]. The exact binomial test estimated confidence intervals (CI) for proportions, with a 95% confidence level. Analyses were performed with StatLib and IBM SPSS 20 software for Windows.

## Results

Out of 69 foxes, 10 (14.5%; CI: 7.2-25.0%) were found infected with *A. platys*; and two (2.9%; CI: 0.4-10.1%) with *E. canis*. Out of all the 78 foxes, one (1.3%; CI: 0.03-6.9%) was infected with *L. infantum* (Table 2). The prevalence of *A. platys* was significantly different from the prevalence of *E. canis* and from that of *L. infantum* (*p*=0.016 and *p*=0.002, respectively). The prevalence values of *E. canis* and *L. infantum* were not statistically different from each other (*p*=0.489).

**Table 2:** **PCR and sequencing for detection of** ***Anaplasma*** **spp.,** ***Ehrlichia*** **spp. and** ***Leishmania*** **spp. in red foxes (** ***Vulpes vulpes*** **) from Portugal**

**Region/sample**	**PCR and sequencing: no. of positive foxes/no. of foxes tested (%)**		
	***Anaplasma platys***	***Ehrlichia canis***	***Leishmania infantum***
North and centre	9/50 (18.0)	1/50 (2.0)	1/50 (2.0)
Blood	3/50 (6.0)	1/50 (2.0)	1/50 (2.0)
Bone marrow	6/47 (12.8)	0/47 (0.0)	1/47 (2.0)
South	1/19 (5.3)	1/19 (5.3)	0/28 (0.0)
Blood	1/14 (7.1)	1/14 (7.1)	0/14 (0.0)
Bone marrow	0/10 (0.0)	0/10 (0.0)	0/19 (0.0)
Total	10/69 (14.5)	2/69 (2.9)	1/78 (1.3)

*A. platys* was detected in nine foxes from all the five northern districts and in one fox from the southern district of Setúbal. *E. canis* was found in one fox from the northern district of Vila Real and in another fox from the southern district of Évora, and *L. infantum* was detected in one fox from the district of Vila Real. No co-infections were found in any one of the 78 foxes.

Paired blood and bone marrow samples (i.e. from the same animal) were available from 52 foxes (47 from the north and five from the south). The percentage of positive results to *A. platys* was 3.8% in blood and 11.5% in bone marrow, but the difference was statistically not significant (*p*=0.289). Positive results to *E. canis* among paired samples were not compared, as the agent was found only in two blood samples. The fox found infected with *L. infantum* was positive both in blood and bone marrow (1.9%; *p*=1.0).

Due to the discrepancy in the number of paired samples (i.e. blood and bone marrow from the same fox) between the north/centre and south regions of Portugal, statistical comparison of results by geographical region was done between blood and bone marrow separately. With blood, the prevalences of *A. platys* (6.0% vs. 7.1%; *p*=1.0), *E. canis* (2.0% vs. 7.1%; *p*=0.392) and *L. infantum* (2.0% vs. 0.0%; *p*=1.0) were not significantly different between the northern/central and southern districts (respectively). With bone marrow, similarly non-significant differences were found for the prevalences of *A. platys* (12.8% vs. 0.0%; *p*=0.171), *E. canis* (0.0% vs. 0.0%; *p* value not computed) and *L. infantum* (2.0% vs. 0.0%; *p*=1.0).

No statistically significant differences were found between genders for the positive results to *A. platys* (17.4% for female vs. 20.7% for male foxes; *p*=1.0), to *E. canis* (0.0% vs. 3.4%; *p*=1.0) or to *L. infantum* (4.3% vs. 0.0%; *p*=0.442). Likewise, the differences of positive results between age groups were not significant for any one of the three agents: *A. platys* (14.3% for juvenile vs. 20.6% for adult foxes; *p*=1.0), *E. canis* (0.0% vs. 2.9%; *p*=1.0) and *L. infantum* (7.1% vs. 0.0%; *p*=0.292).

The foxes were apparently in a healthy body condition, and gross examination and histopathology findings demonstrated a population that seemed not to suffer from histological abnormalities due to infection with *A. platys*, *E. canis* or *L. infantum* (data not shown). However, some samples showed autolytic alterations, in spite of the attempts to minimize post-mortem effects. Moreover, by PCR and DNA sequencing the parasite *Babesia* cf. *microti* was detected in 63 out of 91 (69.2%; CI: 58.7-78.5%) [34] and *Hepatozoon canis* in 68 out of 90 foxes (75.6%; CI: 64.5-83.2%) [25]. The 91 foxes include the 90 foxes and these further include all the 78 foxes from the present study.

**Figure 1:**
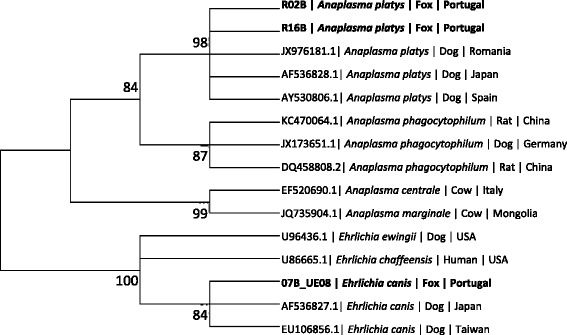
**Maximum likelihood tree phylogram comparing 385 bp sequences of the 16S DNA sequences from** ***Anaplasma*** **spp. and** ***Ehrlichia*** **spp. deposited in GenBank®.** The GenBank® accession numbers, animal source and country of origin from which the sequences were derived are included for each sequence. Bootstrap values higher than 60% are indicated. New sequences derived from the present study are marked in bold letters. The 385 bp sequences included in the phylogenetic analysis were formed by a combination of two non-overlapping 16S rRNA fragments amplified by the E.c-16S fwd/E.c-16S rev primers and EHR16SD/EHR16SR primers. 305 bp DNA sequences included in the phylogram from one fragment of the combined fragment sequences were deposited in GenBank® as accession numbers KP717550, KP717551 (R02B, R16B) for *A. platys* and KP717552 (07B_UE08) for *E. canis*.

The 230 bp DNA sequences of *L. infantum* from the infected Portuguese foxes obtained using the ITS-219 F/ITS-219R primer had 100% identity with the closest GenBank® accession no. GU591397. The DNA sequences of *E. canis* from the infected Portuguese fox had 100% identity with the closest GenBank® accession no. KJ659037. Moreover, sequences from the 16S rRNA fragment amplified by the E.c-16S fwd/E.c-16S rev primers of *Anaplasma* spp. had 100% identity to *A. platys* (KJ659045.1). A longer fragment (303 bp) of the 16S rRNA was amplified from two foxes by the EHR16SD/EHR16SR primers and showed 100% identity with *A. platys* (KM401447). A maximum likelihood tree phylogram based on 385 bp sequences was formed by a combination of the two 16S rRNA fragments amplified by the E.c-16S fwd/E.c-16S rev primers and by the EHR16SD/EHR16SR primers which did not overlap (Figure 1). It indicated that the *Anaplasma* spp. sequences detected in this study from foxes clustered together with *A. platys* sequences deposited in GenBank® and separately from other *Anaplasma* spp., including *A. phagocytophylum. E. canis* sequences from this study clustered with sequences of *E. canis* and other *Ehrlichia* spp. and separately from canine *Anaplasma* spp. (Figure 1). Sequences of pathogens derived from foxes in this study were deposited in GenBank® under the following accession numbers: KP717550, KP717551 (*A. platys*); KP717552 (*E. canis*); and KP738165, KP738166 (*L. infantum*).

## Discussion

Infections with *A. platys*, *E. canis* and *L. infantum* are well documented in domestic dogs, but information on the occurrence of these agents in wild animals is sparse. This is the first known report of *A. platys* in red foxes worldwide, as well as the first molecular evidence of *E. canis* in red foxes from Portugal. In the present study, PCR and subsequent DNA sequencing provided detection and allowed the identification of infectious agents, i.e. *A. platys* and *E. canis*, belonging to genetically close bacterial genera.

The detection of *A. platys* in a moderate proportion of the sampled foxes denotes that these canids are exposed to the agent and suggests that a sylvatic life cycle of this pathogen may exist in vulpine populations in Portugal. *A. platys* appears to be endemic in foxes as it was found in both northern and southern regions of Portugal, and foxes may play a role in the epidemiology of infection with this bacterium and serve as a reservoir for domestic dogs. In addition, the similar prevalence of *A. platys* in foxes from the northern/central and southern regions may be attributable to a relatively homogeneous distribution of the bacterium in its presumed tick vector populations in these areas [35]. In fact, *R. sanguineus* group ticks have been found in mammalian hosts from all the eight districts sampled for the present study [36,37]. The virulence of *A. platys* to red foxes requires further investigation. A non-significant difference between the detection percentage of *A. platys* in blood and bone marrow samples indicates, due to its practicability, that blood is a preferred sample to be collected in living animals. Nevertheless, when sampling corpses it is convenient to obtain samples from more than one tissue, in order to increase the sensitivity of detection [25].

In Portugal, *A. platys* has previously been detected by molecular methods in dogs from the north [22-24], centre [24] and south [24,38,39]. The bacterium has also been detected in *R. sanguineus* group ticks from southern Portugal by PCR and DNA sequencing [35]. Some of the partial *A. platys* 16S rRNA gene sequences obtained from foxes in the present study are most similar to those previously identified in dogs from Spain and Germany, among other countries (Figure 1).

Molecular identification of *E. canis* has been reported in dogs from northeastern [22,23], central [24] and southern Portugal [39,40]; and also in one cat from the south of the country [13]. There are some reports on the presence of antibodies to *E. canis* in red foxes from Europe and the Mediterranean [12,41], but molecular studies on this infection in foxes are more scarce. In central Italy, none of 150 hunted red fox spleens were PCR-positive for *E. canis* DNA [42]. However, in southern Italy the PCR prevalence of this pathogen was 30.8% in 13 red foxes from Sicily [43].

The proven vector of *E. canis* (*R. sanguineus* s.l. [2]), is the most prevalent tick in Portugal and has been found on mammals throughout the country [36,37]. The *E. canis* sequences obtained from the two foxes in this study were identical to the ones previously identified in Portuguese, Spanish and Greek dogs, among sequences from other continents (Figure 1).

The prevalence of *L. infantum* infection detected by means of PCR in the present study (1.3%) is in line with the low level (1.1%) found also by PCR in dogs from southern Portugal [39]. The overall national seroprevalence in dogs has recently been estimated to be 6.3%, with 3.8% in northern Portugal, 5.0% in the district of Setúbal and 2.5% in the district of Évora included in the present fox study [44]. Specific seropositivity in dogs from the municipality of Évora, within the namesake district, was 3.9% in the year 1990, 9.4% in 1999 and 5.6% in 2010 [15]. However, in a close by geographic area of southern Portugal, an indirect immunofluorescence antibody test (IFAT) revealed that 20.4% of 152 dogs had antibodies to *Leishmania*, and PCR detected leishmanial DNA in blood from 34.9% of the same animals [45]. In cats, the molecular prevalence of *L. infantum* in blood was 0.3% out of 320 animals from northern/central [46] and 9.9% in 649 from southern Portugal [13].

In a survey carried out 30 years ago in foxes from the district of Setúbal, 23.0% out of 61 animals tested by an indirect immunofluorescence assay had antibodies to *Leishmania*, and parasites were isolated from tissues of 5.6% of 71 foxes. The prevalence of 5.6% was suggested as sufficient to maintain endemicity of the infection within a semi-autonomic sylvatic cycle in the area [47].

*Leishmania* spp. infection has been reported in red foxes from Italy, France and Spain [48-50]. Different prevalences of fox infection with *Leishmania* have been reported from these countries in southern Europe. The molecular prevalence of *L. infantum* observed in foxes in the present study (1.3%) is much lower than those previously obtained by PCR in blood and spleen samples from the same host species (n=162) in several regions of Spain (14.1% [51]). In the report from Spain, no statistically significant sex or age differences in prevalence were observed in foxes, but there was a significant difference among regions. Interestingly, out of 47 fox serum samples analysed by western blot only one tested positive (2.1%) and DNA restriction patterns differed between red foxes and dogs in two regions [51]. In southern France, among 92 red foxes screened, the PCR prevalence of *L. infantum* was 9%, with DNA found in the spleen and liver, but not in blood, skin or kidney [52]. In southern Italy, 20 (40.0%) out of 50 foxes were PCR-positive simultaneously in lymph node and bone marrow samples, whereas just 17 out of the 20 animals were positive in skin samples [53]. In contrast, Verin et al. [54] failed to find *Leishmania* DNA in red fox skin specimens from central Italy, which may indicate that the parasite had not colonized the tegument of these animals, thereby suggesting that they may only play a minor role as a reservoir in an area that is not highly endemic.

Manifestations of disease in red foxes experimentally infected with *L. infantum* were similar to those found in sick dogs and included skin ulceration, furfuraceous dermatitis, alopecia, weight loss and onychogryphosis [55], but foxes were postulated to have some degree of resistance to *L. infantum* infection [56].

The infection found in the fox in the present study might have been acquired from vectors which fed on dogs, as *L. infantum* is highly prevalent in dog populations in northern Portugal [57]. The phlebotomine sand fly species *Phlebotomus perniciosus* and *Phlebotomus ariasi* are the vectors of *L. infantum* in Portugal [58]. Transmission from foxes to sand flies has not been documented, and studies are necessary to elucidate whether foxes can be infectious to these insects. The role of red foxes as reservoir hosts in the epidemiological cycle of *L. infantum* in Portugal seems to be secondary or even minimal compared to that of dogs. In effect, it is difficult to determine whether fox populations are able to maintain *Leishmania* infection by themselves in the absence of dogs, and this should be studied further.

Altogether, the differences between reports of infection with *L. infantum* from other parts of southern Europe may represent distinct levels of prevalence by geographical location, but may also be due to the distinct assays used, such as serology indicating exposure or PCR detecting infection, or to the different tissues sampled [25]. The different findings on prevalences between *A. platys* and *E. canis* in foxes in the present study could derive from a lower susceptibility of red foxes to the latter, or could be related to a heterogeneous distribution of the agents within the vector populations [35]. In Portugal, where red foxes are abundantly distributed throughout rural and periurban environments, they can serve as a possible reservoir of some pathogens for domestic pets and also to human beings [21,34,43]. However, further studies are needed to evaluate the role of foxes in the epidemiology of *A. platys*, *E. canis* and *L. infantum* in Europe and the Mediterranean area.

## Conclusions

In conclusion, the present study demonstrates that infection with *A. platys* is prevalent in red fox populations in Portugal, whereas *E. canis* and *L. infantum* are present but less frequent. The moderate prevalence of *A. platys* in red foxes suggests that they might be a reservoir of this bacterium and a source of infection to dogs.
